# Circulating Levels of Short-Chain Fatty Acids during Pregnancy and Infant Neurodevelopment

**DOI:** 10.3390/nu14193946

**Published:** 2022-09-23

**Authors:** Carmen Hernández-Martínez, Josefa Canals, Núria Voltas, Francisco Martín-Luján, Victoria Arija

**Affiliations:** 1 Research Group in Nutrition and Mental Health (NUTRISAM), Universitat Rovira i Virgili, 43204 Reus, Spain; 2 Research Center for Behavioral Assessment (CRAMC), Universitat Rovira i Virgili, 43003 Tarragona, Spain; 3 Pere Virgili Institute for Health Research (IISPV), Universitat Rovira i Virgili, 43201 Reus, Spain; 4 Serra Húnter Fellow, Department of Psychology, Faculty of Education Sciences and Psychology, Universitat Rovira i Virgili, 43007 Tarragona, Spain; 5 Research Support Unit Tarragona, Institut Universitari d’Investigació en Atenció Primària Jordi Gol (IDIAP JGol), 43202 Reus, Spain

**Keywords:** short chain fatty acids, acetic acid, propionic acid, butyric acid, isobutyric acid, pregnancy, neurodevelopment, cognitive development, infant

## Abstract

Background: Short-chain fatty acids (SCFA) play a key role in the gut microbiota–brain crosstalk regulating the main neurodevelopmental processes during pregnancy. The aim of this study is to investigate the longitudinal relationship between prenatal levels of the main SCFAs in maternal serum and infant cognitive development and temperament on day 40 postpartum after adjusting for several pre-, peri- and post-natal confounders. Methods: A sample of 357 healthy mother–infant pairs were followed from the beginning of pregnancy to 40 days after birth. Serum SCFA concentrations were assessed in the first and third trimester of pregnancy by LC-MS/MS; and socio-demographic, nutritional, and psychological variables were collected. At 40 days, the Bayley Scales of Infant Development-III and the Early Infancy Temperament Questionnaire were administered. Results: Lower serum levels of acetic, butyric and isobutyric acid, mainly during the first trimester, were related to better language and psychomotor development and, in the case of butyric acid, better intensity behavior in infants. Medium levels of propionic acid were related to better scores for development, mood and temperament. Conclusions: These findings suggest that in a community sample of healthy pregnant women from a Mediterranean region of northern Spain, lower serum levels of SCFAs, especially in the first trimester of pregnancy, seem to be related to better infant neurodevelopment

## 1. Introduction

The gut microbiota is the ecological community of symbiotic and pathogenic microorganisms present in the gut, some of which are critically involved in gut–brain communication [1]. Short-chain fatty acids (SCFAs) are the main metabolites produced by the gut microbiota when it assimilates dietary fiber and protein. Early childhood is a dynamic time for gut colonization and brain development, but little is known about the relationship between these two processes [2]. Both the gut and the brain undergo rapid changes during pregnancy and the early postnatal period, and disruption during these early colonization processes can precipitate a cascade of repercussions that may lead to behavioral and cognitive impairments in infants [3]. The relationship between the mother’s microbiota during gestation and the infant’s brain, behavior and cognitive development has been studied very little and most evidence comes from animal models. These models suggest that gut colonization by microbiota has become integrated into the programming of brain development and interacts in key developmental periods that affect infants’ synaptic activity, cognitive processes, motor activity and social and anxiety-related behaviors [4,5]. In fact, research on SCFAs during pregnancy and early life in animal models suggests that mother-derived SCFAs may pass through the placenta, exposing the fetus at key developmental periods and influencing critical neurodevelopmental processes such as differentiation, neurosphere formation, differentiation from embryonic stem cells into neural cells, neural proliferation and microglia maturation and functioning [2,6,7,8,9,10]. Recent studies on humans have shown that the diversity of maternal fecal microbiota in the third trimester of pregnancy is related to fewer internalizing symptoms in their infant at two years old [11], meaning that the mother’s prenatal microbiome is more relevant to infant neurodevelopment than the infant’s own microbiome [12]. Studying the relationship between circulating serum SCFA levels can be a good approach because the amount of SCFAs can vary considerably in different tissues and only a small fraction of gut-derived SCFAs reaches the systemic circulation [13,14]. However, no studies examining human prenatal SCFA circulating serum levels and its relationship with infant neurodevelopment have been found.

The main SCFAs are acetic, propionic and butyric acid, which make up 90% of all SCFAs. There are others, however, such as isobutyric acid that represent 5% of all SCFAs [15,16]. Emerging evidence shows that SCFAs such as butyric acid stimulate brain-derived neurotrophic factor (BDNF) expression, neurogenesis, and facilitate long-term memory consolidation [7,10,17,18,19]. Additionally, although propionic acid is the least understood SCFA, its activity is known to be associated with beneficial health effects [16] and to play an important role in the development of the brain, cognition and behavior [4,10,20]. In this regard, studies on animals have shown that a prenatal low-fiber diet is related to decreased propionate and butyrate and to offspring with impaired general locomotor activity and increased anxiety-related behaviors [21]. On the basis of these results, it appears that inducing low propionate and butyrate through a low-fiber diet may be harmful. However, other animal studies have shown that rodents prenatally exposed to exogenous injections of propionate also showed impaired memory and learning, altered locomotor activity and stereotyped social and anxiety-related behaviors [22,23,24]. Therefore, although there is still little evidence relating to the function of SCFAs during pregnancy and neurodevelopment, it seems that both low and high levels can affect these processes. Despite these animal studies and even having studied the effect of several levels of SCFAs administered prenatally or modified indirectly by controlling the mother’s diet, whether there is a direct relationship between circulating serum levels of SCFAs during pregnancy and neurodevelopment and infant behavior [4,10] in animals or humans is still unclear. Therefore, since animal studies have shown that SCFAs exert widespread influence on key neurological and behavioral processes and may be involved in critical phases of neurodevelopment, the main aim of this study is to investigate, for the first time in humans, the relationship between circulating maternal blood levels of the main SCFAs (acetic, propionic, butyric and isobutyric) in the first and third trimester of pregnancy and infant cognitive development and temperament on day 40 postpartum, after adjusting for several potential pre-, peri- and post-natal confounders that may also affect infant behavior and neurodevelopment.

## 2. Materials and Methods

### 2.1. Design and Procedure

This is a prospective follow-up study of pregnant women from their first trimester of pregnancy up until 40 days postpartum. It forms part of the ECLIPSES study, a community randomized controlled trial (RCT) conducted in the province of Tarragona (Catalonia, Spain) between 2013 and 2017. The study aimed to determine the highest level of effectiveness of iron supplementation adapted to hemoglobin (Hb) levels in early pregnancy, which would be optimum for mother–child health [25,26]. A total of 793 participants (mean age 30.6 ± 5.1 years) were recruited by midwives at their primary care centers before the 12th week of gestation and were monitored during pregnancy (in the 24th and 36th weeks) and then again on day 40 after birth. Of the 793 participants, 502 attended the postpartum visit.

For participants to be included, they had to be at least 18 years old, have been pregnant for no longer than 12 weeks, not have anemia (Hb > 110 g/L), understand either of the autonomous region’s official languages (Spanish or Catalan), and comply with the study’s requirements. Participants were excluded from the study if they had a multiple pregnancy; had an adverse obstetric history; had taken iron supplements of >10 mg every day during the three months prior to the 12th week of gestation; had reported prior severe illness (state of immunosuppression) or chronic disease that could affect their nutritional status (cancer, diabetes, etc.), or if they had reported liver disease.

Blood samples and sociodemographic, clinical and psychosocial information were collected. Circulating SCFA levels were measured in the 12th and 36th week of gestation in a subsample of 450 participants (selected from among those with no missing data). Of these 450 participants, 357 attended the postpartum visit where their babies were assessed, so the final sample studied consisted of 357 mother–infant pairs. There are no significant differences in SCFA levels or the variables assessed in the study between those participants who attended or did not attend the postpartum visit. The only variable in which significant differences were found was socioeconomic status (SES), with more families in the low-to-medium bracket attending the postpartum visit.

The study was designed in accordance with the Declaration of Helsinki, including the Note of Clarification added in Tokyo in 2004. All procedures involving human subjects were approved by the Clinical Research Ethics Committee of the Jordi Gol Institute in Primary Care Research (IDIAP), the Pere Virgili Institute for Health Research (IISPV) (155/2017) and the Spanish Agency of Medicines and Medical Devices (AEMPS). Informed consent was obtained from all the women who participated in the study. This clinical trial was registered at www.clinicaltrialsregister.eu with EudraCT number 2012-005480-28 and at www.clinicaltrials.gov with identification number NCT03196882.

Table 1 shows the study’s design, the variables collected and the participants.

### 2.2. Instruments and Data Collection

#### 2.2.1. Measurements

Serum samples were collected from pregnant women who had been fasting. The serum was centrifuged and stored at −80 °C until the analysis. Serum concentrations of acetic, propionic, butyric and isobutyric acid were measured by LC-MS/MS [27]. In brief, 20 µL of serum was mixed with an internal standard mixture in methanol to precipitate the proteins. Supernatants were mixed with water, o-Benzylhydroxylamine (BHA, Sigma–Aldrich) and N-(3-Dimethylaminopropyl)-N′-ethylcarbodiimide (EDC, Sigma Aldrich, St. Louis, MO, USA) to obtain SCFA derivatives. The SCFA derivatives were purified by liquid-liquid extraction using diethyl ether and analyzed by ultra-high performance liquid chromatography–mass spectrometry (UHLC-MS/MS) using the UHPLC 1290 Infinity II Series coupled to a QqQ 6470 Series^®^ (Agilent Technologies Inc., Santa Clara, CA, USA). Chromatographic separation was performed with a gradient elution using a ternary mobile phase containing water, methanol and isopropanol with ammonium formate on the analytical Kinetex Polar C18 (2.6 μm 2.1 × 100 mm) column (Phenomenex, Torrance, CA, USA). The mass spectrometer operated in multiple reaction monitoring (MRM) mode and SCFAs were ionized by positive electrospray. The UHPLC-MS/MS system was monitored by the Agilent MassHunter^®^ Workstation (Agilent Technologies Inc., Santa Clara, CA, USA). The validation of the method for quantifying SCFAs in serum is shown in Supplementary Table S1. More detailed information about the SCFAs in our sample can be found in Martún-Grau et al. [28].

Infants’ cognitive development was assessed using the Bayley Scales of Infant Development (BSID-III) [29]. This is an individually administered examination that evaluates the current developmental functioning of infants from 0 to 42 months old. It consists of three general scales (cognitive scale, language scale and motor scale) and four subscales (expressive language, receptive language, fine motor and gross motor). At 40 days old, the cognitive scale assesses infant sensorimotor development and visual attention and exploration. Two subscales make up the language scale: the receptive language subscale assesses an infant’s receptive communication such as pre-verbal behavioral abilities, sound differentiation and social and object orientation; and the expressive language subscale assesses an infant’s pre-verbal communication, such as smiles and early vocalizations. The motor scale comprises the fine motor subscale, which assesses an infant’s prehension, perceptual-motor integration, visual object tracking and response to tactile information, and the gross motor subscale assesses an infant’s movement of limbs and torso, static positioning and balance. Two trained psychologists administered the BSID-III at the visit held on day 40 postpartum. After receiving training on how to jointly administer the test, they achieved a high level of agreement (98%) in the results. All parents were present at the assessments.

The infants’ temperament was assessed using the Early Infancy Temperament Questionnaire (EITQ) [30]. The EITQ is a 76-item questionnaire answered by the infants’ parents for assessing the nine New York Longitudinal Study temperament traits in one-to four-month-old infants. The temperament traits under assessment are: activity level (the level and extent of motor activity), rhythmicity (the degree of regularity of functions such as eating, elimination and the cycle of sleeping and wakefulness), approach (the response to a new object or person in terms of whether the infant accepts the new experience or withdraws from it), adaptability (the adaptability of behavior to changes in the environment), threshold (the sensitivity to environmental stimuli), intensity (the energy level of responses), mood (the infants’ general mood or ‘disposition’, whether cheerful or prone to crying, pleasant or fussy, friendly or unfriendly), distractibility (the degree of the child’s distractibility from what he/she is doing) and persistence (the infant’s attention span and his/her persistence in an activity). A total score is obtained for each temperament trait, which categorizes infant behavior along a continuum from easy/more desirable characteristics (lower score) to difficult/less desirable characteristics (higher score).

#### 2.2.2. Adjustment Measurements

The quality of the pregnant women’s diet was estimated at the 12th and 36th week of gestation according to their adherence to the Mediterranean diet and by using a modified scale from the original Mediterranean diet score (rMED) [31]. The rMED score was obtained by using the Food Intake Frequency Questionnaire (FIFQ) [32] and following the steps given below. The FIFQ contains 45 food and beverage items and the pregnant women were asked about their intake of each of them per week or per month. Each item’s gram per day intake was calculated and they were divided into nine groups: fruit (fruits, nuts and seeds, but not fruit juices), vegetables, legumes, cereals (whole-grain and refined flour, pasta, rice, other grains and bread), fresh fish (fish and seafood), olive oil, total meat (fresh meat and processed meat) dairy products (milk, yogurt, cheese and cream-based desserts) and alcoholic beverages. Each group was expressed in grams per 1000 kcal/day and tertiles were obtained so that participants were assigned a categorical score of low, medium, or high intake (0, 1 or 2, respectively). To obtain the total rMED score, the nine scores were added together. Taking into account the fact that six groups scored positively (fruit, vegetables, legumes, cereals, fresh fish and olive oil) and three components scored negatively (total meat, dairy products, and alcohol), the total rMED score ranged from 0 points (minimal adherence to Mediterranean diet) to 18 points (maximum adherence to Mediterranean diet). The FIFQs were conducted by trained midwives and nutritionists who oversaw the reviewing, the entering of the food data and the data scrubbing and analysis.

Anxiety symptoms during pregnancy were assessed in the first and third trimester by the Spanish version of the State-Trait Anxiety Inventory (STAI) [33]. The STAI is a questionnaire featuring 40 items that assesses state anxiety (the level of transient and situational anxiety) and trait anxiety (the level of dispositional and stable trait anxiety). The state anxiety score was used for this study.

Smoking during pregnancy was assessed by the Fagerström questionnaire (Fagerström_Q) [34]. Women were then classified as smokers or non-smokers according to this information.

The women’s socioeconomic status (SES) was estimated with the Hollingshead index [35] by combining data about the mother’s and her partner’s (if she had one) level of education and profession, classified according to the Catalan classification of occupations [36] in order to obtain a total score representing the family SES.

Mother–infant postpartum bonding difficulties were assessed by the Parent Stress Index-Short Form [37], a 36-item questionnaire that measures the stress directly associated with parenting. A total parent-child dysfunctional interaction score can be obtained.

Data for obstetric and neonatal variables were collected from each woman’s obstetric medical records. These variables included the mother’s age at the beginning of her pregnancy, the mother’s body mass index at each prenatal visit, the infant’s sex, mode of delivery, neonatal birth weight (measured with SECA electronic scales accurate to 10 g), gestational age at birth (verified by ultrasound in obstetric examinations) and infant feeding at the moment of the temperament assessment.

### 2.3. Statistical Analysis

We performed descriptive analyses of the general characteristics of mothers and infants. Differences by trimester of pregnancy were assessed using the chi-squared test for categorical variables and the repeated-measures *t*-test (paired *t*-test) for continuous variables.

Tertiles of the levels of acetic, propionic, butyric and isobutyric acid in the first and third trimester were calculated to obtain groups of SCFA levels at each point. We performed ANCOVA analyses in order to assess the differences in infant cognitive development scores and temperament scales according to the SCFA tertile group during the first and third trimester. The adjustment variables we used were: the mother’s age (in years); the family’s socioeconomic status (total score); first/third trimester state anxiety (total score); smoking during pregnancy (yes, no); first/third trimester quality of diet (total score); first/third trimester mother BMI, mode of delivery (instrumental, non-instrumental); gestational age at birth (weeks); the infant’s weight at birth (in grams); the infant’s sex (girl, boy); breastfeeding on day 40 postpartum (yes, no) and bonding difficulties on day 40 postpartum (total score). We obtained the estimated adjusted means and used the Sidak posthoc analysis to assess significant differences between SCFA level groups.

## 3. Results

### 3.1. General Descriptive Data of the Sample

Table 2 shows the social, prenatal and perinatal descriptive characteristics of the sample. According to these data, the pregnant women had a mean age of 30 years old, 65% were categorized into the medium socioeconomic status and 85% did not smoke during pregnancy. Regarding the birth, the gestational age and the birth weight had normal means, and 66.9% of women had non-instrumental deliveries. Regarding the quality of the mothers’ diets, it tended to increase from the first trimester (mean = 9.58, SD = 2.7) to the third trimester (mean = 9.85, SD = 2.6) (Paired T = 1.953, *p* = 0.050) while state anxiety increased significantly (first trimester mean = 17.33, SD = 8.0; third trimester mean = 18.97, SD = 7.7; Paired T = −5.164, *p* < 0.001).

### 3.2. General Descriptive Data of the Levels of Short Chain Fatty Acids

Table 3 shows the total mean scores of SCFAs per tertile and trimester of pregnancy. The paired T test between the total level of acetic, propionic, butyric and isobutyric acids for the first and third trimester shows that the mean scores are stable throughout the pregnancy except for the butyric acid, which significantly increased from the first (mean = 0.8, SD = 0.1) to the third (mean = 0.9, SD = 0.4) trimester (paired T = −5.080, *p* < 0.001).

### 3.3. Cognitive Development and Temperament for Levels of Acetic, Propionic, Butyric and Isobutyric acid during the First and Third Trimester of Pregnancy

**Figure 1:**
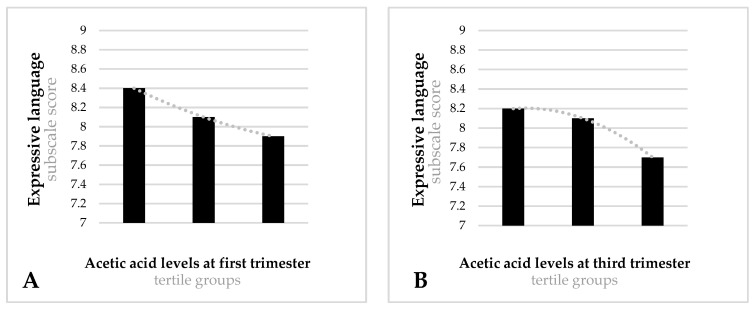
Expressive language subscale scores for tertile groups of acetic acid in the first (**A**) and third (**B**) trimester. (**A**) Low-tertile mean score = 8.4 (SD = 1.5), medium-tertile mean score = 8.1 (SD = 1.5), high-tertile mean score = 7.9 (SD = 1.8). Model F = 3.308; *p* = 0.038. (**B**) Low-tertile mean score = 8.2 (SD = 1.5), medium-tertile mean score = 8.1 (SD = 1.6), high-tertile mean score = 7.7 (SD = 1.5). Model F = 3.543; *p* = 0.030. Models adjusted for mother’s age, family’s socioeconomic status, first/third trimester state anxiety, smoking during pregnancy, first/third trimester quality of diet, first/third trimester BMI, mode of delivery, gestational age at birth, infant’s birth weight, infant’s sex, breastfeeding at 40 days postpartum, and bonding difficulties.

**Figure 2:**
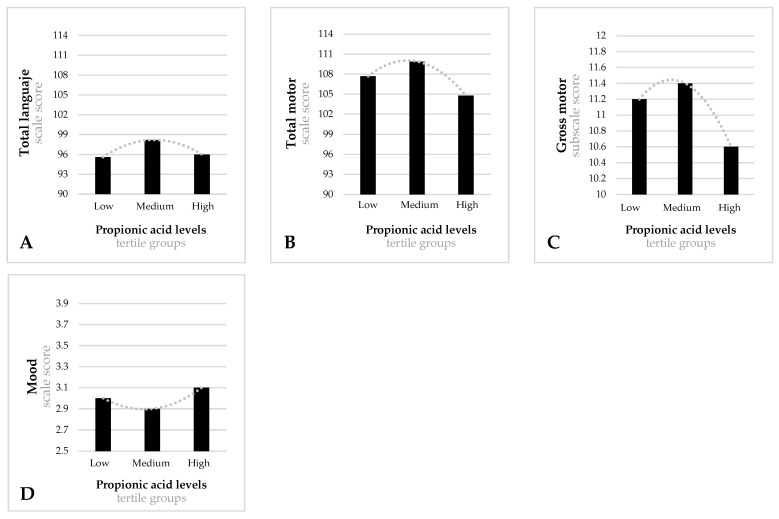
Total language (**A**) and motor scale (**B**), gross motor subscale (**C**), and mood temperament scale (**D**) scores for tertile groups of propionic acid at the first trimester. (**A**) Low-tertile mean score = 97.7 (SD = 8.2), medium-tertile mean score = 98.3 (SD = 8.4), high-tertile mean score = 95.8 (SD = 8.0). Model F = 3.188; *p* = 0.033. (**B**) Low-tertile mean score = 108.4 (SD = 10.9), medium-tertile mean score = 109.6 (SD = 9.6), high-tertile mean score = 104.5 (SD = 13.6). Model F = 5.731; *p* = 0.004. (**C**) Low-tertile mean score = 11.3 (SD = 2.4), medium-tertile mean score = 11.4 (SD = 2.1), high-tertile mean score = 10.6 (SD = 2.4). Model F = 4.342; *p* = 0.014. (**D**) Low-tertile mean score = 3.0 (SD = 0.7), medium-tertile mean score = 2.8 (SD = 0.6), high-tertile mean score = 3.1 (SD = 0.7). Model F = 2.975; *p* = 0.038. Models adjusted for mother’s age, family’s socioeconomic status, first trimester state anxiety, smoking during pregnancy, first trimester quality of diet, first trimester BMI, mode of delivery, gestational age at birth, infant’s birth weight, infant’s sex, breastfeeding at 40 days postpartum, and bonding difficulties.

**Figure 3:**
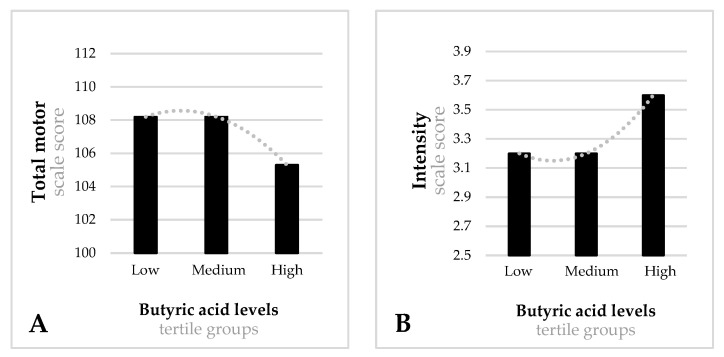
Total motor scale (**A**) intensity temperament scale (**B**) for tertile groups of butyric acid at the first trimester. (**A**) Low-tertile mean score = 109.1 (SD = 10.3), medium-tertile mean score = 108.1 (SD = 10.2), high-tertile mean score = 105.2 (SD = 13.9). Model F = 3.061; *p* = 0.048. (**B**) Low-tertile mean score = 3.3 (SD = 0.8), medium-tertile mean score = 3.2 (SD = 0.9), high-tertile mean score = 3.6 (SD = 0.9). Model F = 6.294; *p* = 0.002. Models adjusted for mother’s age, family’s socioeconomic status, first trimester state anxiety, smoking during pregnancy, first trimester quality of diet, first trimester BMI, mode of delivery, gestational age at birth, infant’s birth weight, infant’s sex, breastfeeding at 40 days postpartum, and bonding difficulties.

**Figure 4:**
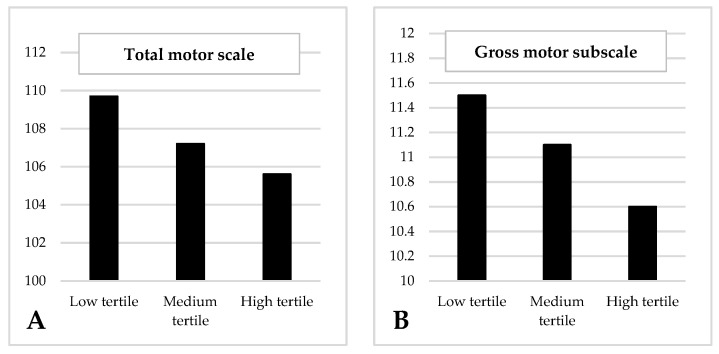
Total motor scale (**A**) and gross motor subscale (**B**) for tertile groups of isobutyric acid at the first trimester. (**A**) Low-tertile mean score = 109.9 (SD = 10.2), medium-tertile mean score = 107.3 (SD = 13.6), high-tertile mean score = 105.4 (SD = 10.7). Model F = 3.722; *p* = 0.025. (**B**) Low-tertile mean score = 11.5 (SD = 2.3), medium-tertile mean score = 11.1 (SD = 2.3), high-tertile mean score = 10.6 (SD = 2.2). Model F = 4.824; *p* = 0.009. Models adjusted for mother’s age, family’s socioeconomic status, first trimester state anxiety, smoking during pregnancy, first trimester quality of diet, first trimester BMI, mode of delivery, gestational age at birth, infant’s birth weight, infant’s sex, breastfeeding at 40 days postpartum, and bonding difficulties.

Figure 1, Figure 2, Figure 3 and Figure 4 show significant differences in the mean scores for cognitive development and temperament between the tertile groups of acetic, propionic, butyric and isobutyric acid.

As regards acetic acid (Figure 2), infants of mothers in the low-tertile group showed better scores on the expressive language subscale (mean = 8.4, SD = 1.5) than infants of mothers in the high-tertile group (mean = 7.8, SD = 1.8) (F = 2.308, *p* = 0.038) (Figure 2A). Similar results were found during the third trimester. Infants of mothers in the low-tertile group showed better mean scores on the expressive language scale (mean = 8.2, SD = 1.5) than infants of mothers in the high-tertile group (mean = 7.7, SD = 1.5) (F = 3.543, *p* = 0.030) (Figure 2B).

As regards propionic acid (Figure 2) during the first trimester, infants of mothers in the medium-tertile group performed significantly better on the total language scale (mean = 98.3, SD = 8.4) (F = 3.188, *p* = 0.033) than infants of mothers in the low-tertile group (mean = 95.7; SD = 8.2) (Figure 2A). Infants of mothers in the medium-tertile group also performed better on the gross motor scale (mean = 11.3, SD = 2.4) (Figure 2B) and total motor scale (mean = 108.4, SD = 9.6) (Figure 2C) than infants of mothers in the high-tertile group (mean = 10.6, SD = 2.4; mean = 104.5; SD = 13.6, respectively) (F = 5.731, *p* = 0.004; F = 4.342, *p* = 0.014, respectively). Similar associations were found on the mood scale (Figure 2D) after the temperament assessment: infants of mothers in the medium-tertile group obtained significantly better scores (mean = 2.8, SD = 0.6) than infants of mothers in the high-tertile group (mean = 3.1, SD = 0.7) (F = 2.975, *p* = 0.038).

As regards butyric acid (Figure 3) during the first trimester, infants of mothers in the low-tertile group performed better on the total motor scale (mean = 109.1, SD = 10.3) than infants of mothers in the high-tertile group (mean = 105.2, SD = 13.9) (F = 3.061, *p* = 0.048) (Figure 3A). In terms of temperament (Figure 3B), infants of mothers in the low- and medium-tertile groups during the first trimester obtained better scores on the intensity scale (mean = 3.3, SD = 0.8; mean = 3.2, SD = 0.9, respectively) than infants of mothers in the high-tertile group (mean = 3.6, SD = 0.9) (F = 6.294, *p* = 0.002).

As regards isobutyric acid (Figure 4) during the first trimester, infants of mothers in the low-tertile group performed significantly better on the gross motor scale (mean = 11.5, SD = 2.3) (Figure 4A) and the total motor scale (Mean = 109.91, SD = 10.2) (Figure 4B) than infants of mothers in the high-tertile group (mean = 10.6, SD = 2.2; mean = 105.4; SD = 10.7, respectively) (F = 4.824, *p* = 0.009; F = 3.722, *p* = 0.025, respectively).

No significant results were found between groups during the third trimester in terms of propionic, butyric and isobutyric levels.

All the means and estimated means of cognitive development scores and infant temperament scales for tertile group levels of acetic, propionic, butyric and isobutyric acids during the first and third trimester are shown in Supplementary Tables S2–S5.

Bearing in mind the sample size of the SCFA tertile groups, and the mean scores and standard deviations of BSID-III and EITQ, and assuming an alpha error of 5%, the statistical power of our results ranges from 70.15% to 95.4%.

## 4. Discussion

This is a follow-up study carried out in a cohort of healthy pregnant women from a Mediterranean region in northern Spain. For the first time in humans, it provides much-needed data on the relationships between maternal circulating serum levels of the main SCFAs (acetic, propionic, butyric and isobutyric) during the first and third trimester of pregnancy and early infant neurodevelopment.

Although the most recent evidence has shown that SCFAs play a major role in achieving and maintaining good gestational health [38] as well as in early neurodevelopment [10], to date no studies in humans have assessed the relationship between circulating serum levels of SCFAs and infant neurodevelopment and behavior. Neither are there any reference ranges or solid data on the levels considered to be optimal for health, so studying tertiles makes it possible to determine whether, within these levels, higher or lower levels can influence neurodevelopment. Previous studies on the relationships between SCFAs and neurodevelopment induced changes in SCFAs by means of infusions or diet modifications but did not assess the final level of SCFAs, the resulting SCFAs in body fluids such as feces, or the metabolites capable of producing SCFAs. This means that it is difficult to compare results because detectable physiological levels in the population may depend on types of body fluid (for example, blood, cerebrospinal fluid, breastmilk, feces, and urine) and health conditions [10]. Therefore, studies that provide data on circulating serum levels of SCFAs are particularly important at this time.

Our results show that infants of mothers in the low-tertile group of acetic acid showed better scores on the expressive language subscale than infants of mothers in the high-tertile group in both the first and third trimester; infants of mothers in the low-tertile group of butyric and isobutyric acid showed better motor development in the first trimester and, in the case of butyric acid, better scores on the intensity temperament scale than infants of mothers in the high-tertile group. In the case of propionic acid, language, motor development and mood are all better in infants of mothers in the medium-tertile group. Although the comparison with animal studies is complicated, in general, our results seem to agree with previous studies which found that the offspring of pregnant dams with induced higher levels of propionic acid showed altered locomotor activity and more anxiety-related behaviors [10,22,23,24,39]. However, our results seem to be inconsistent with the results of the study by Yu et al. [21], which found a relationship between a prenatal low-fiber diet, decreased levels of propionic and butyric acid, and impaired cognitive function and anxiety-related behaviors in offspring. However, their study did not assess the direct relationship between SCFAs and offspring behavior, so it cannot be known to what extent the cognitive and behavioral alterations detected were due to the levels of propionic or butyric acid. On the other hand, the higher mood and intensity scores found in the present study, also in the high tertile of propionic and butyric acid, may indicate a trait of excitability, irritability and negative emotionality [30] which predisposes infants to an increased risk of developing emotional problems [3]. These results contrast with the data provided by Dawson et al. [11], who found that, independently of confounding factors, mothers of children with lower emotionality scores at age two showed higher levels of specific butyrate-producing organisms in the third trimester, although they did not directly assess butyric acid levels. Overall, it is generally accepted that SCFAs have a beneficial effect on brain development, although an overproduction can be detrimental [40]. In this regard, research has shown that higher levels resulting from maternal dysbiosis could have a neuroinflammatory effect after passing the blood–brain barrier, which contributes to a higher risk of cognitive and behavioral problems in infants [8,10,23]. In fact, some studies have shown that they can have detrimental effects on an infant’s host metabolism have been found by other studies (for example, they induce high levels of maternal prenatal butyrate) [41]. Therefore, as Serino [40] reflects, the available data show that SCFAs have the beneficial effects described, but also that the line separating their beneficial and pathological effects is very thin.

As far as pregnancy is concerned, our results support the idea that the beginning of pregnancy is a key period with important neurodevelopment milestones that when disrupted can precipitate a cascade of repercussions that may lead to behavioral and cognitive impairments in the infant [42]. In this regard, while it is still developing, a young fetus’ central nervous system (CNS) may interact with SCFAs produced by the mother’s blood as they cross the barrier of the placenta, and with SCFAs in the amniotic fluid and placenta [10,43,44]. This could have a considerable influence on CNS function, and disrupt neurotransmitter production, mitochondrial function, immune activation and gene expression [8], which are also in early stages of development [10]. What is more, epidemiological and interventional studies have shown that there is a relationship between a mother’s diet, diabetes mellitus, obesity and weight gain during pregnancy and infant neurodevelopment [45,46,47,48,49]. It is plausible that these findings, in addition to being produced by a neuroinflammatory effect, are also mediated by the effects of SCFAs [21,50], since there is a relationship between maternal obesity and weight gain during pregnancy and higher levels of acetic acid in humans [51], and prenatal maternal weight gain and obesity are associated with a higher risk of behavioral alterations [52]. In this sense, Liu et al. performed a set of animal experiments regarding prenatal obesity and offspring neurodevelopment and found that maternal preconception induced-obesity were related to offspring deficits in cognition and social behavior, and that maternal high-fiber intake applied in obese pregnant dams improves the offspring’s cognitive and behavioral deficits [50]. Considering that a high-fiber diet may produce higher SCFA levels, these data in from animal models are not consistent with our results but suggest a role between diet-microbiota-SCFA and neurodevelopment, independently of the mother’s prenatal obesity. Therefore, among many others, obesity and diet should be important factors to consider when studying the relationship between the maternal microbiota, the metabolites produced by it, and infant early neurodevelopmental processes.

The limitations of our study should be borne in mind when interpreting the results. First, as we have no reference values, we have studied the relationships by tertile analyses, which provide less precise information. Moreover, the statistical power is, in some cases, lower than 80%, a value that is not generally regarded as acceptable. Nevertheless, we measured SCFAs in maternal circulation at two points during pregnancy in a large sample of healthy pregnant woman and have provided innovative data in humans; and the relationship between SCFAs and infant neurodevelopment has been adjusted for a considerable number of pre-, peri- and post-natal sociodemographic, psychological and obstetrical confounders that have previously been related to infant neurodevelopment, such as prenatal smoking exposure, quality of pregnant diet and BMI increase, thus helping us to fine-tune the relationships under study. Although more research in human community samples is needed to clarify the role SCFAs have on early neurodevelopment, our results allow us to conclude that in a healthy Mediterranean community sample, lower circulating levels of SCFAs (and medium levels in the case of propionic acid) at the beginning of pregnancy are associated with better language and motor development scores in infants, and with more desirable temperament characteristics at 40 days old. Considering that the onset of gestation is when SCFAs have a significant effect on fetal neurodevelopment, it would be very interesting to make a detailed study of the relationship between diet, healthy habits, circulating serum SCFAs and neurodevelopment during pregnancy so that specific recommendations and assessments can be made, not only when women become pregnant, but also when they are planning to become pregnant so that they can conceive with the best possible health indicators and ensure the best possible health of mothers-to-be and their infants.

## Figures and Tables

**Table 1:** Study design. Sample and variables collected.

	12 Weeks of Gestation	36 Weeks of Gestation	Partum	40 Days Postpartum
Participants	450	450	450	357
**Individual assessment**				- **Neurodevelopment** (Bayley Scales for Infant Development—cognitive scale—language scale (expressive—receptive)—motor scale (fine—gross))
**Medical records**	- Body Mass Index - Mother age	- Body Mass Index	- Gestational age at birth - Infant weight - Mode of delivery	- Infant breastfeeding
**Questionnaires**	- Quality of diet (Mediterranean diet adherence—Food Intake Frequency Questionnaire) - Smoking during pregnancy (Fagerström questionnaire) - Anxiety symptoms (State Trait Anxiety Inventory)	- Quality of diet (Mediterranean diet adherence—Food Intake Frequency Questionnaire) - Smoking during pregnancy (Fagerström questionnaire) - Anxiety symptoms (State Trait Anxiety Inventory)		- **Temperament** (Early Infancy Temperament Questionnaire—activity level—rhythmicity - approach—adaptability - threshold—intensity—mood—distractibility—persistent)
**Blood samples**	- **Short Chain Fatty Acids** (Acetic-propionic—butyric—isobutyric)	- **Short Chain Fatty Acids** (Acetic-propionic—butyric—isobutyric)		
**Family Interview**	- Socioeconomic status (Hollingshead index)			

**Table 2:** Sociodemographic and perinatal descriptive variables.

	Mean (SD) * ***n*(%) ^#^**
Mother’s age (years)	30.6 (5.1) *
Family’s socioeconomic status	
Low	84 (16.8) ^#^
Medium	323 (64.7) ^#^
High	92 (18.4) ^#^
Mother smoking during pregnancy (no)	428 (85.8) ^#^
Quality of diet (total score)	
1st trimester	9.6 (2.7)
3rd trimester	9.9 (2.6)
Paired T (p)	1.953 (0.051)
State anxiety (total score)	
1st trimester	17.3 (8.0)
3rd trimester	19.0 (7.7)
Paired T (p)	−5.164 (<0.001)
Infant sex (girls)#	251 (50.5) ^#^
Mode of delivery (non-instrumental)	340 (66.9) ^#^
Gestational age (weeks)	39.70 (1.4) *
Birth weight (gr)	3278.3 (460.9)
Infant feeding (breastfeeding)	407 (81.9) ^#^
Mother–infant bonding (total score)	17.32 (7.9) *
**Bayley Scales Infant Development**	**Mean (SD)**
Cognitive scale (total score)	101.8 (8.8) *
Language scale (total score)	96.4 (8.4) *
Receptive (total score)	10.6 (2.1) *
Expressive (total score)	8.1 (1.6) *
Motor scale (total score)	107.9 (11.2) *
Fine (total score)	11.5 (1.9) *
Gross (total score)	11.1 (2.3) *
**Early Infant Temperament Questionnaire**	**Mean (SD)**
Activity level (total score)	3.2 (0.7) *
Rhythmicity (total score)	3.3 (0.8) *
Approach (total score)	2.6 (0.7) *
Adaptability (total score)	2.6 (0.7) *
Intensity (total score)	3.3 (0.9) *
Mood (total score)	3.0 (0.7) *
Persistence (total score)	3.0 (0.8) *
Distractibility (total score)	2.6 (0.8) *
Threshold (total score)	4.1 (0.7) *

* Data are shown as mean (SD); ^#^ Data are shown as n (%).

**Table 3:** Mean SCFA scores per tertile and trimester of pregnancy.

	First Trimester				Third Trimester				Paired T (*p*) *
	Low-Tertile Mean (SD)	Medium-Tertile Mean (SD)	High-Tertile Mean (SD)	Total Sample * Mean (SD)	Low-Tertile Mean (SD)	Medium-Tertile Mean (SD)	High-Tertile Mean (SD)	Total Sample * Mean (SD)	
Acetic acid	30.9 (7.7)	45.7 (3.6)	70.1 (22.5)	49.1 (21.4)	32.5 (5.7)	45.2 (3.1)	67.4 (18.1)	48.5 (18.3)	0.439 (0.661)
Propionic acid	2.7 (0.3)	3.5 (0.2)	4.5 (0.6)	3.6 (0.9)	2.6 (0.3)	3.3 (0.2)	4.6 (0.9)	3.5 (1.0)	0.385 (0.701)
Butyric acid	0.5 (0.1)	0.7 (0.1)	1.2 (0.3)	0.8 (0.3)	0.5 (0.1)	0.8 (0.1)	1.4 (0.4)	0.9 (0.4)	−5.080 (<0.001)
Isobutyric acid	0.3 (0.1)	0.5 (0.1)	0.7 (0.2)	0.5 (0.2)	0.3 (0.1)	0.4 (0.1)	0.7 (0.3)	0.4 (0.2)	1.635 (0.103)

* Paired T: Mean differences between total sample mean scores of first trimester and third trimester.

## Data Availability

Not applicable.

## Supplementary Materials

The following supporting information can be downloaded at: https://www.mdpi.com/article/10.3390/nu14193946/s1, Table S1: Method validation for quantification of SCFA in serum; Table S2: Cognitive and temperament mean and adjusted mean scores according to acetic acid levels in first and third trimester; Table S3: Cognitive and temperament means and adjusted means scores according to propionic acid levels in first and third trimester; Table S4: Cognitive and temperament means and adjusted means scores according to butyric acid levels in first and third trimester; Table S5: Cognitive and temperament means and adjusted means scores according to isobutyric acid levels in first and third trimester.
