# Supplementary material for: Circulating Levels of Short-Chain Fatty Acids during Pregnancy and Infant Neurodevelopment
Source: Nutrients. 2022 Sep 23;14(19):3946. doi: 10.3390/nu14193946 (PMC9573109; doi:10.3390/nu14193946)
Supplement: Supplementary file 1 [file nutrients-14-03946-s001.zip › nutrients-1885422-supplementary.pdf]

Supplementary Table S1. Method validation for quantification of SCFA in serum

	AMR/Linearity (μmol/L)	LOQ (μmol/L)	LOD (μmol/L)	Precision (CV%)		Calibration curve	
				Intra-day	Inter-day	Slope	r^2
Acetic acid (C2:0)	0.011 – 1828	0.011	0.003	4.9	11.1	1.067	0.998
Propionic acid (C3:0)	0.004 – 101	0.004	0.001	8.8	15.2	0.051	0.999
Isobutyric acid (C4:0)	0.005 – 85	0.005	0.002	4.3	9.8	0.006	0.997
Butyric acid (C4:0)	0.005 – 68	0.005	0.002	4.3	10.5	0.007	0.999

AMR: Analytical measurement range; LOQ: limit of detection; LOQ: limit of quantification.

Supplementary Table S2. Cognitive and temperament mean and adjusted mean scores according to acetic acid levels in first and third trimester

		First trimester					Third trimester				
**Bayley Scales Infant Development**		**Low tertile[a] < 39.686 μM**	**Medium tertile[b] 39.687-51.972 μM**	**High tertile[c] > 51.972 μM**	**F (p)**	**Sidak**	**Low tertile[a] < 39.636 μM**	**Medium tertile[b] 39.637 - 50.667 μM**	**High tertile[c] > 50.667 μM**	**F (p)**	**Sidak**
Cognitive scale	Mean (SD)	101.8 (8.5)	102.4 (8.0)	101.6 (7.7)	0.184 (0.832)		101.0 (7.8)	101.8 (7.7)	101.9 (8.0)	0.655 (0.520)	
	Estimated mean	101.7	102.3	101.9			100.8	102.0	101.9		
Language scale	Mean (SD)	97.4 (8.2)	97.2 (7.2)	95.2 (9.3)	1.887 (0.153)		97.4 (7.5)	96.6 (8.4)	94.6 (8.2)	2.833 (0.061)	
	Estimated mean	97.3	97.2	95.4			97.4	96.6	94.6		
Receptive	Mean (SD)	10.7 (2.0)	11.0 (2.0)	10.5 (2.3)	1.012 (0.364)		10.9 (1.9)	10.7 (2.1)	10.5 (2.2)	0.712 (0.491)	
	Estimated mean	10.7	10.9	10.5			10.8	10.7	10.5		
Expressive	Mean (SD)	8.4 (1.5)	8.1 (1.5)	7.8 (1.8)	3.308 (0.038)	ac (0.031)	8.2 (1.5)	8.1 (1.6)	7.7 (1.5)	3.543 (0.030)	ac (0.042)
	Estimated mean	8.4	8.1	7.9			8.2	8.1	7.7		
Motor scale	Mean (SD)	108.2 (10.4)	106.3 (13.6)	107.7 (10.5)	0.872 (0.458)		106.7 (14.5)	108.6 (10.5)	107.1 (10.4)	0.937 (0.393)	
	Estimated mean	107.8	106.3	108.1			106.4	108.7	107.3		
Fine	Mean (SD)	11.5 (1.8)	11.4 (2.1)	11.5 (1.9)	0.180 (0.835)		11.4 (2.0)	11.6 (2.1)	11.4 (1.7)	0.284 (0.753)	
	Estimated mean	11.4	11.4	11.6			11.4	11.6	11.5		
Gross	Mean (SD)	11.2 (2.4)	10.9 (2.1)	11.0 (2.4)	0.581 (0.560)		11.0 (2.3)	11.2 (2.4)	11.0 (2.3)	0.349 (0.706)	
	Estimated mean	11.2	10.9	11.1			11.0	11.3	11.0		
**Early Infant Temperament Questionnaire**											
Activity level	Mean (SD)	3.2 (0.7)	3.2 (0.7)	3.2 (0.7)	0.073 (0.929)		3.1 (0.7)	3.1 (0.7)	3.2 (0.6)	0.345 (0.708)	
	Estimated mean	3.2	3.1	3.2			3.2	3.1	3.2		
Rhythmicity	Mean (SD)	3.3 (0.8)	3.4 (0.7)	3.2 (0.7)	1.906 (0.150)		3.2 (0.8)	3.3 (0.8)	3.2 (0.8)	0.211 (0.810)	
	Estimated mean	3.3	3.4	3.2			3.2	3.3	3.2		
Approach	Mean (SD)	2.6 (0.7)	2.6 (0.7)	2.6 (0.7)	0.058 (0.944)		2.7 (0.8)	2.5 (0.7)	2.7 (0.7)	2.460 (0.087)	
	Estimated mean	2.7	2.6	2.6			2.7	2.5	2.7		
Adaptability	Mean (SD)	2.6 (0.6)	2.6 (0.7)	2.6 (0.7)	0.111 (0.895)		2.6 (0.7)	2.6 (0.6)	2.6 (0.7)	0.131 (0.877)	
	Estimated mean	2.6	2.6	2.6			2.6	2.6	2.6		
Intensity	Mean (SD)	3.3 (0.8)	3.4 (0.8)	3.4 (1.0)	0.826 (0.439)		3.4 (0.9)	3.2 (0.8)	3.4 (0.8)	1.703 (0.184)	
	Estimated mean	3.3	3.3	3.4			3.4	3.2	3.4		
Mood	Mean (SD)	2.9 (0.7)	3.1 (0.7)	2.9 (0.6)	2.598 (0.076)		2.9 (0.7)	3.0 (0.7)	2.9 (0.7)	0.643 (0.291)	
	Estimated mean	2.9	3.1	2.9			3.0	3.0	2.9		
Persistence	Mean (SD)	2.9 (0.8)	2.9 (0.7)	3.0 (0.8)	0.130 (0.878)		2.9 (0.8)	2.9 (0.8)	3.0 (0.7)	1.265 (0.284)	
	Estimated mean	2.9	2.9	2.9			2.9	2.9	3.0		
Distractibility	Mean (SD)	2.5 (0.8)	2.6 (0.7)	2.5 (0.8)	0.964 (0.382)		2.5 (0.7)	2.6 (0.8)	2.5 (0.8)	0.225 (0.798)	
	Estimated mean	2.6	2.6	2.5			2.5	2.6	2.5		
Threshold	Mean (SD)	4.1 (0.7)	4.2 (0.7)	4.1 (0.6)	0.172 (0.842)		4.1 (0.7)	4.1 (0.6)	4.2 (0.7)	0.074 (0.929)	
	Estimated mean	4.1	4.2	4.2			4.1	4.1	4.2		

Models adjusted by mother's age, family's socioeconomic status, first/third trimester state anxiety, smoking during pregnancy, first/third trimester quality of diet, first/third trimester BMI, mode of delivery, gestational age at birth, infant's birth weight, infant's sex, breastfeeding at 40 days postpartum, and bonding difficulties.

Supplementary Table S3. Cognitive and temperament means and adjusted means scores according to propionic acid levels in first and third trimester

		**First trimester**					**Third trimester**				
**Bayley Scales Infant Development**		**Low tertile[a] < 3.120 μM**	**Medium tertile[b] 3.121 - 3.837 μM**	**High tertilec > 3.837 μM**	**F (p)**	**Sidak**	**Low tertile[a] < 3.033 μM**	**Medium tertile[b] 3.034 - 3.663 μM**	**High tertile[c] >3.663 μM**	**F (p)**	**Sidak**
Cognitive scale	Mean (SD)	101.6 (7.8)	102.7 (8.3)	101.2 (8.0)	1.297 (0.275)		101.4 (8.2)	101.9 (8.0)	101.6 (7.8)	0.100 (0.905)	
	Estimated mean	101.4	102.9	101.4			101.4	101.6	101.9		
Language scale	Mean (SD)	95.7 (8.2)	98.3 (8.4)	95.8 (8.0)	3.188 (0.033)	ab (0.040)	96.4 (8.2)	96.8 (8.7)	95.3 (7.1)	1.352 (0.260)	
	Estimated mean	95.6	98.2	96.0			96.3	97.1	95.2		
Receptive	Mean (SD)	10.5 (2.0)	11.0 (2.0)	10.5 (2.2)	1.765 (0.173)		10.8 (2.0)	10.8 (2.2)	10.5 (2.0)	0.529 (0.590)	
	Estimated mean	10.5	11.0	10.6			10.8	10.8	10.5		
Expressive	Mean (SD)	7.97 (1.6)	8.37 (1.6)	8.03 (1.5)	2.038 (0.132)		7.97 (1.5)	8.15 (1.5)	7.85 (1.6)	1.510 (0.233)	
	Estimated mean	8.0	8.4	8.0			7.9	8.2	7.8		
Motor Scale	Mean (SD)	108.4 (10.9)	109.6 (9.6)	104.5 (13.6)	5.731 (0.004)	bc (0.003)	108.1 (11.0)	108.2 (10.0)	105.9 (14.5)	0.808 (0.447)	
	Estimated mean	107.7	109.9	104.8			108.3	107.9	106.2		
Fine	Mean (SD)	11.5 (2.0)	11.8 (1.9)	11.2 (1.9)	2.449 (0.088)		11.6 (2.0)	11.5 (1.9)	11.3 (2.0)	0.225 (0.799)	
	Estimated mean	11.3	11.8	11.3			11.6	11.4	11.4		
Gross	Mean (SD)	11.3 (2.4)	11.4 (2.1)	10.6 (2.4)	4.342 (0.014)	bc (0.014)	11.1 (2.5)	11.2 (2.0)	11.0 (2.5)	0.084 (0.919)	
	Estimated mean	11.2	11.4	10.6			11.1	11.2	11.0		
**Early Infant Temperament Questionnaire**											
Activity level	Mean (SD)	3.2 (0.7)	3.2 (0.7)	3.2 (0.7)	0.060 (0.942)		3.1 (0.7)	3.2 (0.7)	3.2 (0.7)	1.426 (0.242)	
	Estimated mean	3.2	3.2	3.1			3.1	3.2	3.2		
Rhythmicity	Mean (SD)	3.3 (0.8)	3.2 (0.8)	3.3 (0.8)	1.214 (0.299)		3.2 (0.8)	3.3 (0.8)	3.3 (0.8)	1.074 (0.343)	
	Estimated mean	3.3	3.2	3.3			3.2	3.3	3.2		
Approach	Mean (SD)	2.6 (0.7)	2.6 (0.8)	2.7 (0.7)	1.540 (0.216)		2.6 (0.7)	2.6 (0.7)	2.7 (0.8)	0.866 (0.422)	
	Estimated mean	2.6	2.7	2.7			2.6	2.6	2.7		
Adaptability	Mean (SD)	2.6 (0.6)	2.5 (0.7)	2.7 (0.7)	0.959 (0.384)		2.5 (0.7)	2.6 (0.6)	2.7 (0.7)	1.281 (0.279)	
	Estimated mean	2.6	2.5	2.7			2.5	2.6	2.7		
Intensity	Mean (SD)	3.3 (0.7)	3.3 (0.9)	3.4 (0.9)	1.244 (0.290)		3.3 (0.8)	3.4 (0.8)	3.4 (0.8)	0.253 (0.777)	
	Estimated mean	3.3	3.3	3.4			3.3	3.4	3.4		
Mood	Mean (SD)	3.0 (0.7)	2.8 (0.6)	3.1 (0.7)	2.975 (0.038)	bc (0.037)	2.9 (0.7)	3.0 (0.6)	3.1 (0.6)	2.092 (0.125)	
	Estimated mean	3.0	2.9	3.1			2.9	3.0	3.1		
Persistence	Mean (SD)	3.0 (0.9)	2.8 (0.7)	3.0 (0.8)	2.468 (0.086)		2.9 (0.8)	3.0 (0.85)	3.0 (0.8)	0.097 (0.908)	
	Estimated mean	3.0	2.8	3.0			2.9	3.0	3.0		
Distractibility	Mean (SD)	2.6 (0.8)	2.4 (0.8)	2.6 (0.8)	0.565 (0.569)		2.5 (0.7)	2.5 (0.7)	2.6 (0.8)	0.595 (0.552)	
	Estimated mean	2.5	2.5	2.6			2.5	2.6	2.6		
Threshold	Mean (SD)	4.1 (0.7)	4.1 (0.6)	4.2 (0.6)	0.989 (0.373)		4.1 (0.6)	4.1 (0.8)	4.2 (0.6)	0.079 (0.924)	
	Estimated mean	4.1	4.1	4.2			4.1	4.1	4.2		

Models adjusted by mother's age, family's socioeconomic status, first/third trimester state anxiety, smoking during pregnancy, first/third trimester quality of diet, first/third trimester BMI, mode of delivery, gestational age at birth, infant's birth weight, infant's sex, breastfeeding at 40 days postpartum, and bonding difficulties.

Supplementary Table S4. Cognitive and temperament mean and adjusted mean scores according to butyric acid levels in first and third trimester.

		First trimester					Third trimester				
**Bayley Scales Infant Development**		**Low tertile[a] < 0.613 μM**	**Medium tertile[b] 0.614-0.862 μM**	**High tertile[c] >0.862 μM**	**F (p)**	**Sidak**	**Low tertile[a] <0.682 μM**	**Medium tertile[b] 0.683-0.971 μM**	**High tertile[c] >0.972 μM**	**F (p)**	**Sidak**
Cognitive scale	Mean (SD)	102.4 (6.7)	102.5 (8.3)	100.8 (8.0)	1.452 (0.236)		102.1 (7.5)	100.5 (9.0)	101.8 (7.4)	0.456 (0.634)	
	Estimated mean	102.3	102.5	100.9			102.0	100.9	101.6		
Language scale	Mean (SD)	96.8 (8.1)	96.8 (7.9)	96.5 (8.8)	0.003 (0.997)		97.4 (8.0)	95.3 (8.4)	95.8 (7.8)	1.568 (0.210)	
	Estimated mean	96.7	96.7	96.7			97.3	95.4	95.7		
Receptive	Mean (SD)	10.7 (2.0)	10.8 (2.1)	10.6 (2.3)	0.134 (0.875)		11.0 (2.0)	10.5 (2.2)	10.5 (1.9)	1.390 (0.251)	
	Estimated mean	10.7	10.8	10.7			10.9	10.6	10.5		
Expressive	Mean (SD)	8.2 (1.7)	8.1 (1.5)	8.2 (1.5)	0.140 (0.870)		8.1 (1.5)	7.9 (1.6)	8.0 (1.6)	0.776 (0.461)	
	Estimated mean	8.2	8.1	8.2			8.1	7.9	8.0		
Motor scale	Mean (SD)	109.1 (10.3)	108.1 (10.2)	105.2 (13.9)	3.061 (0.048)	ac (0.050)	107.6 (9.7)	107.4 (10.9)	107.5 (15.2)	0.003 (0.997)	
	Estimated mean	108.8	108.2	105.3			107.5	107.5	107.6		
Fine	Mean (SD)	11.7 (1.8)	11.6 (1.8)	11.2 (2.1)	1.161 (0.314)		11.4 (1.9)	11.6 (1.9)	11.4 (2.1)	0.454 (0.636)	
	Estimated mean	11.6	11.6	11.3			11.4	11.6	11.4		
Gross	Mean (SD)	11.3 (2.3)	11.1 (2.3)	10.8 (2.4)	1.255 (0.286)		11.1 (2.1)	10.9 (2.4)	11.4 (2.4)	1.504 (0.224)	
	Estimated mean	11.3	11.1	10.8			11.0	10.9	11.5		
**Early Infant Temperament Questionnaire**											
Activity level	Mean (SD)	3.1 (0.7)	3.1 (0.7)	3.2 (0.7)	0.953 (0.387)		3.1 (0.7)	3.2 (0.7)	3.2 (0.6)	0.043 (0.958)	
	Estimated mean	3.1	3.1	3.2			3.2	3.2	3.2		
Rhythmicity	Mean (SD)	3.2 (0.7)	3.3 (0.7)	3.3 (0.8)	0.283 (0.754)		3.1 (0.8)	3.3 (0.8)	3.3 (0.7)	1.262 (0.285)	
	Estimated mean	3.2	3.3	3.2			3.2	3.3	3.3		
Approach	Mean (SD)	2.6 (0.7)	2.6 (0.7)	2.6 (0.8)	0.050 (0.952)		2.6 (0.7)	2.6 (0.7)	2.7 (0.7)	0.451 (0.637)	
	Estimated mean	2.7	2.6	2.6			2.6	2.7	2.7		
Adaptability	Mean (SD)	2.6 (0.7)	2.6 (0.7)	2.6 (0.7)	0.077 (0.926)		2.6 (0.6)	2.6 (0.7)	2.6 (0.7)	0.305 (0.737)	
	Estimated mean	2.6	2.6	2.6			2.6	2.6	2.6		
Intensity	Mean (SD)	3.3 (0.8)	3.2 (0.9)	3.6 (0.9)	6.294 (0.002)	ac (0.007)	3.2 (0.9)	3.4 (0.8)	3.4 (0.8)	1.045 (0.353)	
	Estimated mean	3.2	3.2	3.6		bc (0.008)	3.2	3.4	3.4		
Mood	Mean (SD)	2.9 (0.6)	3.0 (0.7)	3.0 (0.7)	1.373 (0.255)		2.9 (0.6)	3.0 (0.7)	3.0 (0.7)	0.482 (0.618)	
	Estimated mean	2.9	3.0	3.0			2.9	3.0	3.0		
Persistence	Mean (SD)	2.9 (0.8)	3.0 (0.8)	2.9 (0.8)	2.853 (0.059)		2.9 (0.8)	2.9 (0.9)	3.0 (0.7)	0.087 (0.916)	
	Estimated mean	2.8	3.1	2.9			2.9	2.9	3.0		
Distractibility	Mean (SD)	2.5 (0.7)	2.5 (0.8)	2.5 (0.8)	0.275 (0.760)		2.5 (0.7)	2.5 (0.8)	2.5 (0.8)	0.065 (0.937)	
	Estimated mean	2.5	2.6	2.5			2.5	2.5	2.5		
Threshold	Mean (SD)	4.1 (0.6)	4.1 (0.6)	4.2 (0.7)	0.769 (0.464)		4.1 (0.6)	4.2 (0.7)	4.1 (0.7)	0.562 (0.571)	
	Estimated mean	4.1	4.1	4.2			4.1	4.2	4.2		

Models adjusted by mother's age, family's socioeconomic status, first/third trimester state anxiety, smoking during pregnancy, first/third trimester quality of diet, first/third trimester BMI, mode of delivery, gestational age at birth, infant's birth weight, infant's sex, breastfeeding at 40 days postpartum, and bonding difficulties.

Supplementary Table S5. Cognitive and temperament mean and adjusted mean scores according to isobutyric acid levels in first and third trimester.

Bayley Scales Infant Development		First trimester Low tertile[a] < 0.390 μM	Medium tertile[b] 0.391-0.507 μM	High tertile[c] >0.507 μM	F (p)	Sidak	Third trimester Low tertilea <0.331 μM	Medium tertile[b] 0.332-0.463 μM	High tertile[c] >0.463μM	F (p)	Sidak
Cognitive scale	Mean (SD)	103.4 (7.3)	101.3 (8.4)	101.3 (8.4)	2.690 (0.069)		101.9 (7.3)	101.0 (8.3)	101.8 (8.4)	0.686 (0.505)	
	Estimated mean	103.4	101.2	101.4			101.9	100.8	102.0		
Language scale	Mean (SD)	97.1 (8.3)	96.6 (8.2)	96.0 (8.3)	0.272 (0.762)		97.2 (7.2)	95.1 (8.9)	96.5 (8.1)	1.220 (0.297)	
	Estimated mean	97.0	96.6	96.2			96.9	95.2	96.6		
Receptive	Mean (SD)	10.7 (2.0)	10.8 (2.2)	10.6 (2.1)	0.246 (0.782)		10.9 (1.9)	10.6 (2.2)	10.7 (2.2)	0.325 (0.723)	
	Estimated mean	10.7	10.8	10.6			10.9	10.6	10.7		
Expressive	Mean (SD)	8.2 (1.6)	8.0 (1.5)	8.1 (1.7)	0.402 (0.669)		8.2 (1.4)	7.7 (1.7)	8.1 (1.5)	1.591 (0.206)	
	Estimated mean	8.2	8.0	8.1			8.1	7.8	8.1		
Motor scale	Mean (SD)	109.9 (10.2)	107.3 (13.6)	105.4 (10.7)	3.722 (0.025)	ac (0.021)	109.0 (14.6)	107.2 (10.0)	106.2 (10.3)	1.286 (0.278)	
	Estimated mean	109.7	107.2	105.6			109.0	107.0	106.5		
Fine	Mean (SD)	11.8 (1.9)	11.5 (1.9)	11.2 (2.1)	1.806 (0.166)		11.8 (1.9)	11.4 (2.0)	11.2 (2.0)	2.404 (0.092)	
	Estimated mean	11.7	11.5	11.2			11.8	11.3	11.3		
Gross	Mean (SD)	11.5 (2.3)	11.1 (2.3)	10.6 (2.2)	4.824 (0.009)	ac (0.006)	11.4 (2.5)	11.0 (2.1)	10.8 (2.2)	1.564 (0.211)	
	Estimated mean	11.5	11.1	10.6			11.4	11.0	10.8		
**Early Infant Temperament Questionnaire**											
Activity level	Mean (SD)	3.1 (0.7)	3.2 (0.7)	3.2 (0.7)	0.277 (0.758)		3.1 (0.7)	3.1 (0.7)	3.3 (0.7)	3.089 (0.050)	
	Estimated mean	3.1	3.2	3.2			3.1	3.1	3.3		
Rhythmicity	Mean (SD)	3.4 (0.8)	3.2 (0.7)	3.2 (0.7)	2.003 (0.137)		3.2 (0.8)	3.3 (0.7)	3.3 (0.8)	0.608 (0.545)	
	Estimated mean	3.4	3.2	3.2			3.2	3.3	3.3		
Approach	Mean (SD)	2.6 (0.7)	2.6 (0.7)	2.7 (0.7)	1.162 (0.314)		2.6 (0.7)	2.7 (0.7)	2.6 (0.7)	1.252 (0.287)	
	Estimated mean	2.6	2.6	2.7			2.7	2.7	2.6		
Adaptability	Mean (SD)	2.5 (0.6)	2.6 (0.7)	2.7 (0.7)	0.418 (0.659)		2.6 (0.7)	2.5 (0.6)	2.7 (0.6)	1.378 (0.254)	
	Estimated mean	2.6	2.6	2.6			2.6	2.5	2.7		
Intensity	Mean (SD)	3.3 (0.8)	3.3 (0.8)	3.4 (1.0)	0.532 (0.588)		3.4 (0.8)	3.3 (0.8)	3.4 (0.9)	0.837 (0.434)	
	Estimated mean	3.3	3.3	3.4			3.4	3.3	3.4		
Mood	Mean (SD)	2.9 (0.7)	3.1 (0.7)	3.0 (0.6)	1.195 (0.304)		3.0 (0.7)	3.0 (0.6)	3.0 (0.7)	0.385 (0.681)	
	Estimated mean	2.9	3.1	3.0			3.0	3.0	3.0		
Persistence	Mean (SD)	2.9 (0.8)	2.9 (0.8)	3.0 (0.8)	1.426 (0.242)		2.9 (0.8)	2.9 (0.8)	3.0 (0.8)	0.278 (0.758)	
	Estimated mean	2.9	2.9	3.0			2.9	2.9	3.0		
Distractibility	Mean (SD)	2.5 (0.8)	2.5 (0.8)	2.5 (0.8)	0.075 (0.928)		2.5 (0.8)	2.5 (0.7)	2.6 (0.8)	0.606 (0.546)	
	Estimated mean	2.5	2.5	2.5			2.5	2.5	2.6		
Threshold	Mean (SD)	4.1 (0.7)	4.1 (0.7)	4.2 (0.6)	0.631 (0.533)		4.2 (0.7)	4.1 (0.6)	4.1 (0.7)	0.422 (0.656)	
	Estimated mean	4.1	4.1	4.2			4.2	4.1	4.2		

Models adjusted by mother's age, family's socioeconomic status, first/third trimester state anxiety, smoking during pregnancy, first/third trimester quality of diet, first/third trimester BMI, mode of delivery, gestational age at birth, infant's birth weight, infant's sex, breastfeeding at 40 days postpartum, and bonding difficulties.
